# 

**Published:** 1898-10

**Authors:** 


### Ohio State Dental Society.

The regular annual meeting of the Ohio State Dental Society will be held in Columbus on the first Tuesday of December next.

The indications are that the meeting will be one of great importance and interest to every progressive dentist in the State, and it is greatly desired that there be as large an attendance as possible.

Each year these State meetings are becoming of more and more importance. This is true because of the progress the profession is making, and because of the reorganization of the National Association. This body is moving forward in its work, and it is desirable that the State societies which are auxiliary to it keep abreast with it.

The Executive Committee give good assurance that there will be much important work for the society. Quite a number of papers are already promised, and the indications are that business matters of great interest will be presented and call for action. Now, we suggest that every member of the society, and every member of the profession in the State, who has real interest in his profession will be present at that time and that each will be prepared to make some contribution that will add to the interest of the occasion.

A good program is being arranged, which will appear in the next number of this journal. Let every one then, from this time forward, so arrange his business matters as to enable him, without fail, to attend the meeting.

THE DENTAL REGISTER,

A MONTHLY MAGAZINE DEVOTED TO THE INTERESTS OF THE

DENTAL PROFESSION.

*Terms Reduced to $2.00 Per Year. Single Copies, 25 Cents.*

EDITED BY PROF. J. TAFT, D.D.S.

—AND—

Published by SAM'L A. CROCKER & CO., Ohio Dental and Surgical Depot.

The volume commences in January and closes in December.

The REGISTER being issued monthly is always fresh, therefore Indispensable to the practitioner who desires to keep posted up with the new inventions and improvements which are daily developed. Neither expense nor effort will be spared to give value and variety to its contents. Articles will be illustrated with well executed engravings whenever they will add to the interest or assist to explanation.

Its extensive circulation and frequency of issue make it one of the BEST DENTAL ADVERTISING MEDIUMS in the United States.

All subscriptions must begin with January or July.

Local or special notices, five lines or less, will be inserted for $3 each insertion; over five lines, 50 cts. per line.

All advertising bills payable in advance, or at furthest, after the issue of the first number containing the advertisement. All transient advertisements to be paid for in advance.

☞CONTRIBUTIONS SOLICITED.

Exclusively Editorial Communications should be addressed to the Editor.

The latest number of the REGISTER may be ordered with or without other good[illegible] any time, and all letters should be addres[illegible]

